# Sex Differences in Fat Distribution and Muscle Fat Infiltration in the Lower Extremity: A Retrospective Diverse-Ethnicity 7T MRI Study in a Research Institute Setting in the USA

**DOI:** 10.3390/diagnostics14202260

**Published:** 2024-10-10

**Authors:** Talon Johnson, Jianzhong Su, Johnathan Andres, Anke Henning, Jimin Ren

**Affiliations:** 1 Advanced Imaging Research Center, University of Texas Southwestern Medical Center, Dallas, TX 75390, USA; anke.henning@utsouthwestern.edu; 2 Department of Mathematics, University of Arlington, Arlington, TX 76019, USA; su@uta.edu; 3 Department of Mathematics, University of Houston, Houston, TX 77004, USA; jaandres@cougarnet.uh.edu; 4 Department of Radiology, University of Texas Southwestern Medical Center, Dallas, TX 75390, USA

**Keywords:** skeletal muscle, fat infiltration, subcutaneous fat, bone marrow, MRI, aging, obesity

## Abstract

**Background:** Fat infiltration in skeletal muscle is related to declining muscle strength, whereas excess subcutaneous fat is implicated in the development of metabolic diseases. **Methods:** Using multi-slice axial T2-weighted (T2w) MR images, this retrospective study characterized muscle fat infiltration (MFI) and fat distribution in the lower extremity of 107 subjects (64M/43F, age 11–79 years) with diverse ethnicities (including White, Black, Latino, and Asian subjects). **Results:** MRI data analysis shows that MFI, evaluated by the relative intensities of the pixel histogram profile in the calf muscle, tends to increase with both age and BMI. However, statistical significance was found only for the age correlation in women (*p* < 0.002), and the BMI correlation in men (*p* = 0.04). Sex disparities were also seen in the fat distribution, which was assessed according to subcutaneous fat thickness (SFT) and the fibula bone marrow cross-sectional area (BMA). SFT tends to decrease with age in men (*p* < 0.01), whereas SFT tends to increase with BMI only in women (*p* < 0.01). In contrast, BMA tends to increase with age in women (*p* < 0.01) and with BMI in men (*p* = 0.04). Additionally, MFI is positively correlated with BMA but not with SFT, suggesting that compromised bone structure may contribute to fat infiltration in the surrounding skeletal muscle. **Conclusions:** The findings of this study highlight a sex factor affecting MFI and fat distribution, which may offer valuable insights into effective strategies to prevent and treat MFI in women versus men.

## 1. Introduction

The complex relationship between body fat distribution and muscle metabolic health has long been a focus in medical research [1]. While it is widely recognized that sex differences exist in fat distribution—women typically accumulate more fat in the hips and thighs, whereas men are more prone to abdominal visceral fat [2]—less is known about how fat is distributed within and surrounding the skeletal muscle of the lower legs. Furthermore, sex variations in this regard remain largely unexplored, despite their potential implications in metabolic regulation and disease development [3,4,5],

Recent studies have highlighted the importance of sex-specific factors in calf fat metabolism [3,6]. For example, subcutaneous fat (SF) tissue thickness (SFT) is notably higher in females with lipedema, a condition affecting mostly women [6]. Additionally, the connection between visceral fat and bone marrow fat (BMF) raises questions about the role of BMF in osteoporosis, a condition that is prevalent among the elderly. BMF increases with age gradually in men, but sharply in women after menopause [7]. Exploring how the bone marrow cross-sectional area (BMA), SFT, and demographic factors such as BMI and age interact, especially across the sexes, is crucial for gaining insights into the dynamics of fat accumulation and distribution.

The harmful accumulation of fat in non-adipose tissues such as the heart, kidney, liver, and skeletal muscle, may cause mitochondria dysfunction. This lipotoxicity may increase the risk of insulin resistance and comorbidities such as obesity, heart disease, stroke, and type 2 diabetes. In the skeletal muscle, fat infiltration contributes to muscle weakness and dysfunction [8,9,10,11]. This process involves the progressive elevation of both intramyocellular lipids (IMCL, in the form of droplets) and extramyocellular lipids (EMCL, including perivascular adipose tissues (PVAT) and intramuscular adipose tissue (IMAT)). MRI has emerged as a sensitive tool for detecting small anatomical alterations [12,13], and muscle fat infiltration (MFI) may serve as a reliable biomarker for monitoring disease progression.

However, challenges remain in quantifying fat content in muscle. Conventional DIXON approaches do not work well in muscle due to the known fiber orientation effects of the resonance frequencies of the lipid NMR signals [8,14,15]. Segmenting pure fat tissue from lean muscle on MRI images is also complicated by the partial volume effect and artifacts from field inhomogeneity [16,17,18,19,20]. The ^1^H MR spectroscopic technique allows for the extraction of specific fat metabolic information from muscle, but unlike MRI it lacks a high spatial resolution and wide anatomical coverage [21].

In this study, by analyzing T2-weighted MRI images from 107 subjects of diverse ethnicities and varying age and BMI, we quantified muscle fat infiltration and assessed fat accumulation in bone marrow and subcutaneous tissues. Our aim was to explore sex-specific differences and demographic factors in SFT and BMA, examine the correlations between these metrics, and investigate their relationship with fat infiltration severity. The findings from this study may provide valuable insights for strategies to combat musculoskeletal and fat metabolic disorders.

## 2. Materials and Methods

### 2.1. Subjects and Data Acquisition

MRI images were acquired from 107 subjects, comprising 43 females aged 52.7 ± 14.6 years (in the range 15–78 years) and 64 males aged 57.9 ± 17.8 years (in the range 11–79 years). These subjects were from diverse ethnic backgrounds (White Caucasians, Black African Americans, Latinos, and Asians), enrolled in clinical studies either as healthy participants or as patients without primary muscle conditions, in the Advanced Imaging Research Center, University of Texas Southwestern Medical Center, USA. The average BMI was 28.4 ± 4.4 kg/m^2^ (in the range 19.0–38.5) for males and 30.3 ± 5.7 kg/m^2^ (in the range 19.7–44.3) for females. The MRI scan protocol was approved by the Institutional Review Board and informed consent was obtained from all participants prior to the scan.

All subjects were positioned feet-first and supine in the MRI scanner (7T Achieva, software release R5.7, Philips Healthcare, Best, The Netherlands), with the calf muscle positioned parallel to the magnetic field and directly on the detection coil (Philips Healthcare). The coil was a partial-volume, double-tuned, ^1^H/^31^P quadrature coil. The center of the coil was positioned approximately one-third of the distance along the leg from the knee to the heel. Nine slices of axial, T2-weighted, turbo-spin echo images were acquired. Typical parameters were as follows: field of view 180 × 180 mm, in-plane spatial resolution 0.7 × 0.7 mm^2^, slice thickness 4 mm, gap 2 mm; repetition time (TR) 2 s, echo time (TE) 75 ms, turbo factor 16, and number of acquisitions (NA) = one, and acquisition time 1.5 min.

### 2.2. Data Processing and Analysis

**Figure 1:**
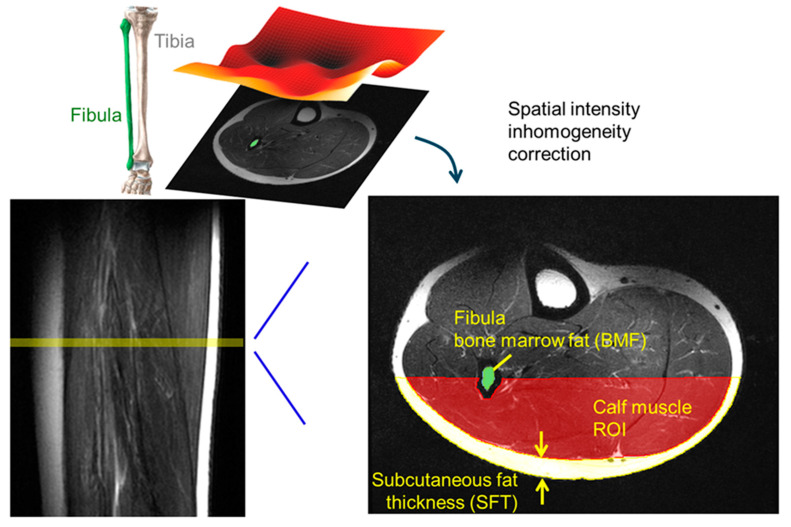
Segmentation of fibula bone marrow (green), subcutaneous fat (yellow), and calf muscle ROI (red) in T2w MR Image. Fat infiltration in calf muscle evaluated by an analysis of the pixel histogram after the correction of spatial intensity inhomogeneity based on the N4ITK algorithm.

MRI image processing and histogram profiling were performed using freely available multi-image analysis GUI (Mango, version 4.1) (https://mangoviewer.com/). Image spatial inhomogeneity correction, also known as bias field correction, was performed using the NT4ITK method available within the SimpleITK 2.4 library for Python 3.9 [22,23,24] (https://simpleitk.org/). This correction was carried out to enhance the accuracy of the histogram analysis of pixel intensities in the region of interest (ROI). Mango’s ROI tools were used to obtain the pixel histogram within the calf muscle ROI and to manually segment the fibula bone marrow and the subcutaneous tissue, as shown in Figure 1. To ensure consistency in ROI analysis and the minimization of spatial inhomogeneity effects (Figure S1, Supplementary Materials), only the periphery calf muscle inferior to the fibula bone in the central seven slices was studied (starting with the second slice), with the ROI upper boundary defined by a straight horizontal line dividing the fibula from its center along the LR direction (Figure 1, right panel). The subcutaneous fat thickness (SFT) was computed by averaging the SFT measurements from the seven central slices. For each slice, the SFT was calculated by dividing the peripheral subcutaneous tissue area (A, in mm^2^) by the central length of the SF tissue curvature, which is the average of the lengths of the inner and outer SF tissue curvature (L1 and L2, in mm), using the following formula:

$$SFT=\frac{1}{7}\sum_{i=1}^{7}\left(\frac{2A_{i}}{{\left(L1+L2\right)}_{i}}\right)$$

where i represents the series number of the selected central slices.

The averaged profile of pixel histograms, computed from the central seven slices, was analyzed by curve fitting using two different line shapes, a symmetric Gaussian and an asymmetric pseudo-Gaussian, with the line width defined by the formula *a* + *b*x, in which *a* and *b* are two fitting constants, and x represents the variable of pixel intensity. The pseudo-Gaussian lineshape becomes Gaussian when *b* = 0. The curve fitting was performed using MATLAB’s *lsqcurvfit* function. To ensure easy comparison of images from different individuals, each histogram profile was normalized to 100 in the integral. Several parameters characterizing the features of the fitted histogram profile were extracted, including mean pixel intensity, mode pixel intensity (at which the pixel count reaches the maximum), skewness (Pearson’s coefficient = (mean − mode)/standard deviation), and the full linewidth (LW) of the histogram profile at half height.

The measurement reproducibility, computed by standard deviation divided by average (Δx/$\bar{x}$), was assessed by five repeated manual ROI segmentations of the same MRI image by the same operator. Then, the resulting measurement variations were used as the input of random noise to evaluate the correlation between the noise-added variables (SFT, BMA, and MFI indexes) and demographic factors (age and BMI). The *p* values of correlations from ten different executions were averaged and compared with the correlations without such noise.

As the study group’s average BMIs (men 28.4, women 30.3) were very close to the cutoff BMI for overweight versus obese (=30.0), we performed a correlation analysis not only for the entire group but also for obese vs. non-obese subgroups for comparison.

### 2.3. Principal Component Analysis (PCA) and Fuzzy C-Means (FCM) Clustering

The features extracted from the muscle pixel distribution profile were subjected to further PCA analysis for dimensionality reduction and feature selection [25]. The input variables to the PCA algorithm included mean pixel intensity, mode pixel intensity, and linewidth. These inputs were standardized using z-score normalization to ensure comparability across features. PCA was then performed on the standardized data to identify the principal components that captured the maximum variance. We determined the number of components necessary to explain at least 95% of the total variance. The significance of each original feature was assessed by summing the absolute values of its coefficients in the selected principal components. Features were ranked by their importance, and the top two were chosen for further analysis.

The PCA-selected features, including both mean and mode pixel intensities, were then employed to categorize the subjects into four subgroups with muscle fat infiltration (MFI) of varying severity using the Fuzzy C-Means (FCM) algorithm [26,27,28]. Both PCA and FCM were performed in MATLAB, using the *pca* and *fcm* functions, respectively. Following the subgroup clustering, an average histogram profile was obtained for each subgroup by population average.

### 2.4. Statistical Analysis

Matlab’s function *ttest2*, the two-sample *t*-test, was performed to test the null hypothesis that two independent measurements have equal means. The test rejects the null hypothesis at the 5% significance level. Matlab’s function *corrcoef* was used to evaluate the linear correlation between two diffident sets of measurements, with a *p*-value < 0.05 representing statistical significance.

## 3. Results

### 3.1. Sex Differences in SFT and BMA

**Figure 2:**
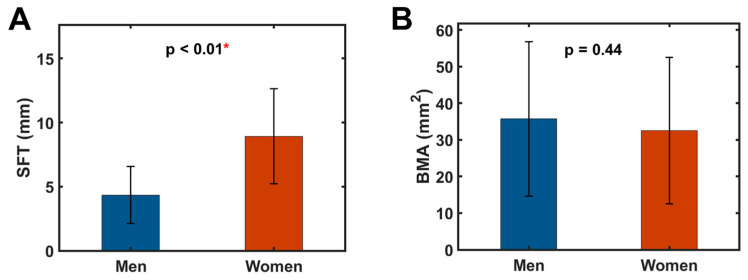
Group average of (**A**) subcutaneous fat thickness SFT and (**B**) bone marrow cross-sectional area BMA in men (*n* = 64) and women (*n* = 43). The symbol * denotes statistical significance.

Figure 1 depicts the manual ROI segmentation of the fibula bone marrow, subcutaneous fat tissue, and the periphery calf muscle. For this group of 107 subjects of diverse ethnicity, age, and BMI, significant sex differences were found in SFT, with females being twice as large as males (8.9 ± 3.7 mm, *n* = 43 vs. 4.3 ± 2.2 mm, *n* = 64, *p* < 0.01, Figure 2A). Unlike SFT, the measured BMA values were similar between men and women (35.7 ± 21.1 mm^2^ vs. 32.6 ± 20.0 mm^2^, *p* = 0.44, Figure 2B).

### 3.2. SFT Correlation with Age and Body Mass Index (BMI)

**Figure 3:**
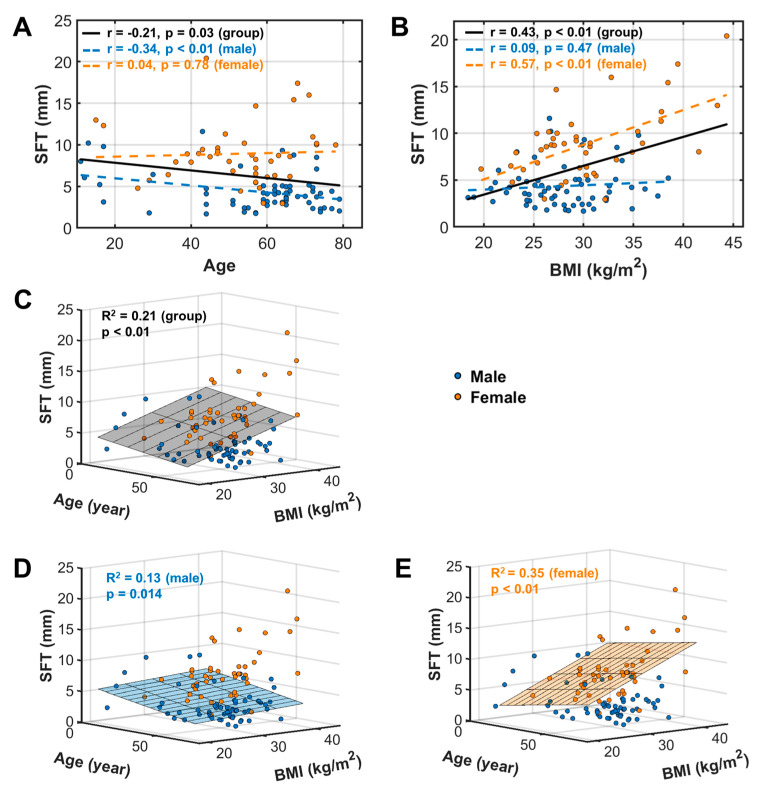
SFT linear univariate correlation with age (**A**) and BMI (**B**), and multivariate correlation with age and BMI for the entire group (**C**), and male (**D**) and female (**E**) subgroups.

Our univariate correlation analysis found that the subcutaneous fat thickness (SFT) was linearly correlated with both age (Figure 3A, *p* = 0.03) and BMI (Figure 3B, *p* < 0.01). For the entire group (*n* = 107), SFT appears to decrease with age but increase with BMI.

Sex subgroup analysis shows that the SFT’s age dependence is statistically significant only in males (*p* < 0.01, *n* = 64, Figure 3A), particularly in the non-obese subgroup (*p* < 0.01, *n* = 69, Figure S2, Supplementary Materials), but not in females (*p* = 0.78, *n* = 43, Figure 3A), whether obese or non-obese (Figure S2, Supplementary Materials). The opposite was true for the SFT-BMI relationship (*p* < 0.01 for females vs. *p* = 0.47 for males, Figure 3B).

A multivariate correlation analysis of SFT with age and BMI yielded significant correlations for the entire group (*p* < 0.01, Figure 3C), as well as the male (*p* = 0.014, Figure 3D) and female (*p* < 0.01, Figure 3E) subgroups.

### 3.3. BMA Correlation between with Age and BMI

**Figure 4:**
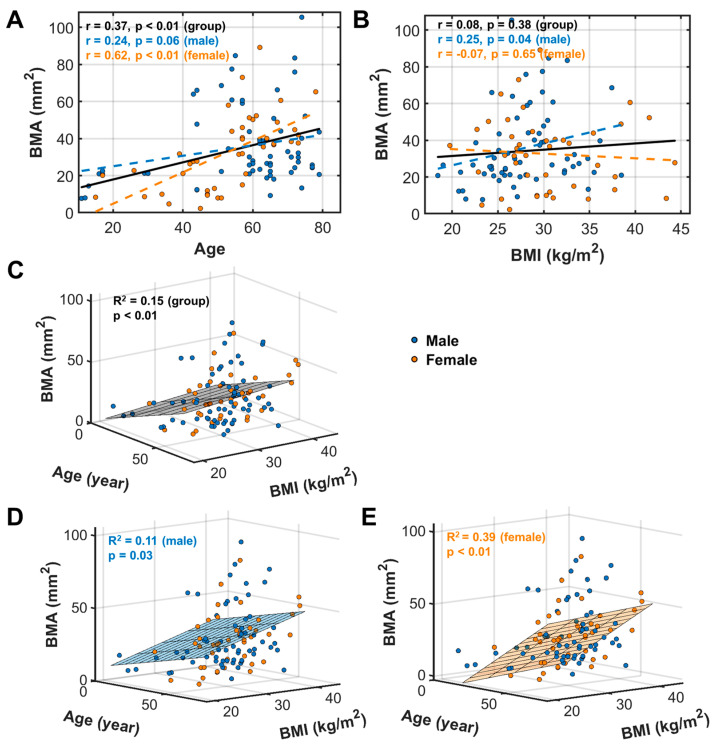
BMA linear univariate correlation with age (**A**) and BMI (**B**), and multivariate correlation with age and BMI for the entire group (**C**), and male (**D**) and female (**E**) subgroups.

Sex differences were also found in the BMA’s correlation with age (Figure 4A) and BMI (Figure 4B). In contrast to SFT, BMA tends to increase with age (Figure 4A). Also, this correlation is significant in females (*p* < 0.01) but not in males (*p* = 0.06), whether obese or non-obese (*p* < 0.01 for females, *p* > 0.05 for males, Figure S3, Supplementary Materials). BMA tends to increase with BMI but only in males (*p* = 0.04) and not in females (*p* = 0.65, Figure 4B). As a group (male + female), BMA was significantly correlated with age (*p* < 0.01, Figure 4A), but not with BMI (*p* = 0.38, Figure 4B).

For the multivariate analysis, BMA was found to be significantly correlated with age and BMI as covariates in the entire group (*p* < 0.01, Figure 4C), as well as in male (*p* = 0.03, Figure 4D) and female (*p* < 0.01, Figure 4E) subgroups. Similar trends in BMA changes were found in terms of age and BMI dependence between the univariate and multivariate analyses (Tables S1 and S2, Supplementary Materials).

### 3.4. Histogram Profile, Muscle Fat Infiltration (MFI), and Fuzzy C-Means (FCM) Clustering

**Figure 5:**
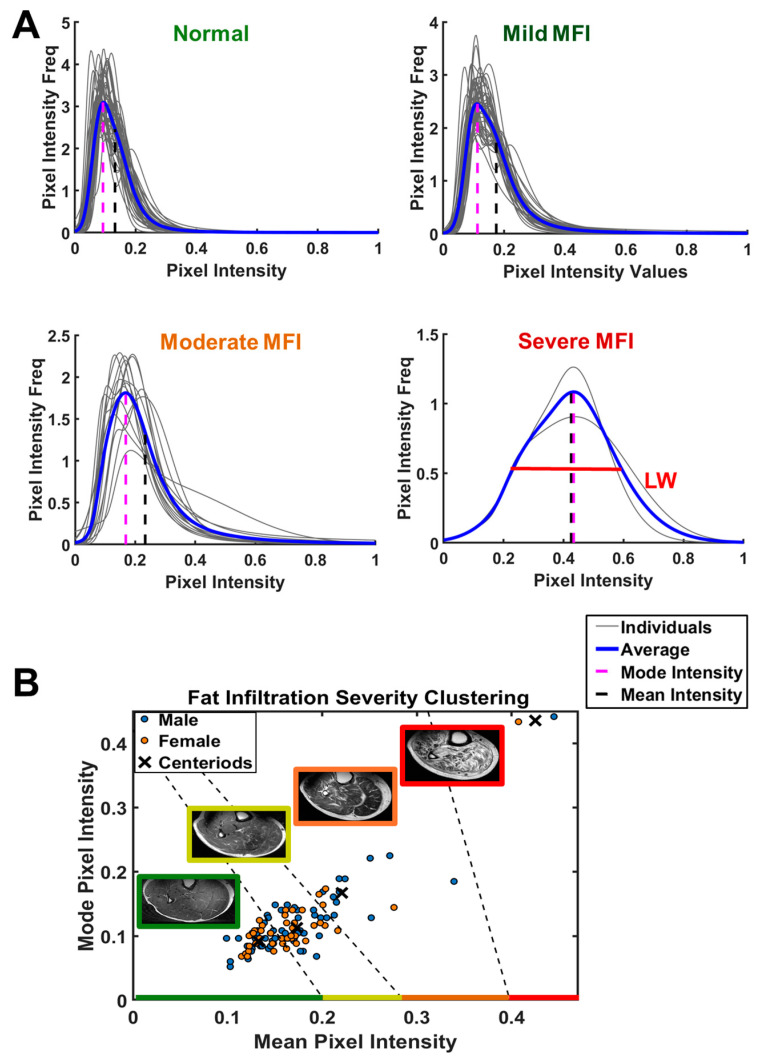
Analysis of pixel histogram for the characterization of the severity of fat infiltration in calf muscle. (**A**) Pixel intensity distribution profiles, showing mean pixel intensity (black dash line) and mode pixel intensity (magenta dash line). (**B**) Subject clustering based on the measurements of pixel mean intensity and mode intensity. Muscle fat infiltration (MFI) in 107 subjects clustered into four groups: normal (45/107, 26M/19F), mild MFI (45/107, 26M/19F), moderate MFI (15/107, 11M/4F), and severe MFI (2/107, 1M/1F). Note the trend in MFI is reflected by the increase in mean intensity, mode intensity, and linewidth (profile dispersion).

Figure 5A shows the pixel histogram profiles obtained from the calf muscle ROI. The mean pixel intensity (blue vertical dash line), the mode pixel intensity (magenta vertical dash line), and the linewidth (LW) of the histogram profile all tend to increase as fat infiltration into the muscle becomes more severe. Using the FCM algorithm, the entire group of subjects (*n* = 107) was clustered into four subgroups based on the MFI severity levels, as measured by the mode and mean pixel intensities. The rationale behind the choice of four subgroups, rather than three or five, was based on two key factors: the clusters’ linear separability [29,30] and the minimization of the objective function solved by the FCM algorithm, with the values of these being 0.096 and 0.056 for three and four subgroups, respectively—both showing linear separability—while the five-subgroup solution, with a value of 0.039, lacked linear separability. 

According to their MFI levels, the four selected subgroups ranged from “normal” as measured by their low mode and low mean pixel intensities, to mild, moderate, and severe MFI with progressive increases in both mode and mean pixel intensities. This can be clearly seen by the increasing trend of muscle marbling appearance in the MRI images (Figure 5B). Of the 107 total subjects, 42% (45/107, 26M/19F) were categorized as normal, 42% (45/107, 26M/19F) as showing mild fat infiltration, 14% (15/107, 11M/4F) as showing moderate fat infiltration, and 2% (2/107, 1M/1F) as showing severe fat infiltration.

Given the apparently high correlation between mean and mode pixel intensities, we also examined clustering with mean pixel intensity alone, which yielded quite similar results, with four groups being identified according to MFI severity, including normal 41% (44/107, 27M/17F), mild MFI 35% (37/107, 20M/17F), moderate MFI 21% (23/107, 15M/7F), and severe MFI 3% (3/107, 2M/1F), as shown in Figure S4, Supplementary Materials.

The inter-group transition appears to be continuous from normal to mild MFI, and from mild to moderate MFI. In contrast, a large gap exists between moderate to severe MFI, with the average mode and mean pixel intensities being twice as high in the severe group (comprising two CKD patients on dialysis) than the other densely-populated groups with lower MFIs (Figure 5). Given this data distribution feature, the MFI data analysis presented hereafter is focused only on those three densely populated subgroups, clustered by both mean and mode pixel intensities.

### 3.5. Characterization of MFI

#### 3.5.1. Features of MFI Index-Clustered Groups

**Figure 6:**
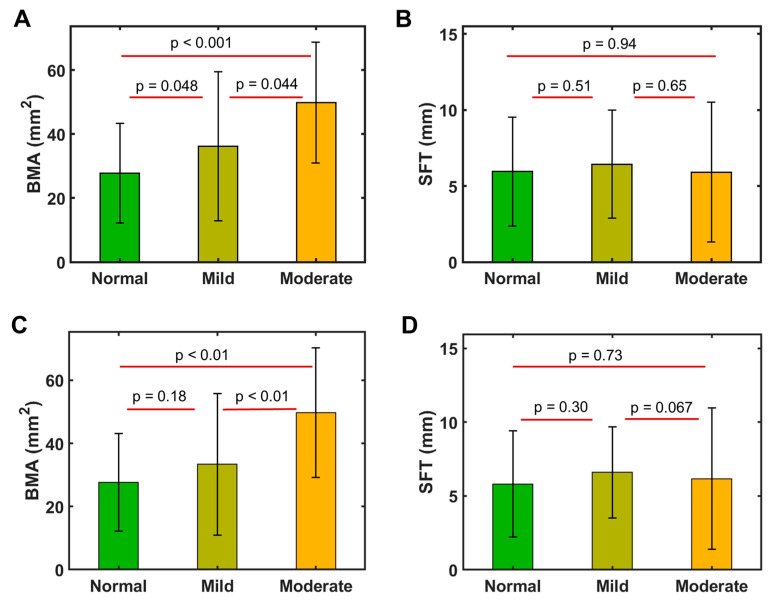
Comparison of the averaged BMA (**A**,**C**) and SFT (**B**,**D**) for those three MFI groups (normal, mild, and moderate), categorized by MFI mean and mode indexes (**A**,**B**) and by mean index alone (**C**,**D**).

For those three major groups, categorized by MFI mean and mode indexes, there is a significant difference in BMA (*p* < 0.05, Figure 6A), which averaged 27.7 ± 15.5 mm^2^ for the normal MFI group, 36.1 ± 23.2 mm^2^ for the mild MFI group, and 27.7 ± 15.5 mm^2^ for the moderate MFI group, respectively. 

In contrast, the average SFT was quite similar between these MFI groups (5.9 ± 3.6 mm for the normal, 6.4 ± 3.5 mm for the mild, and 5.9 ± 4.6 mm for the moderate, *p* > 0.05, Figure 6B).

For those three major groups clustered by MFI mean index alone, a significant difference was seen in BMA but only between the mild and moderate groups (*p* < 0.01, Figure 6C). No inter-group difference was found in SFT (Figure 6D).

#### 3.5.2. MFI Correlation between MFI Indexes with BMA and SFT

**Figure 7:**
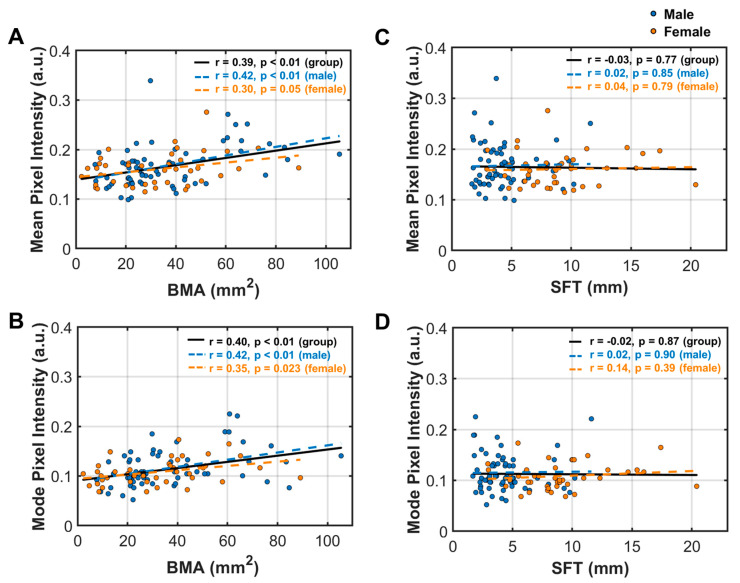
Analysis of linear correlations between muscle fat infiltration (MFI) indexes with BMA and SFT. (**A**) BMA area vs. mean pixel intensity; (**B**) BMA area vs. mode pixel intensity; (**C**) SFT vs. mean pixel intensity; (**D**) SFT vs. mode pixel intensity.

For both men and women, the mean and mode MFI indexes both show significant correlations with BMA (Figure 7A,B) but not with SFT (Figure 7C,D). MFI tends to increase with BMA, with stronger correlations in men than in women (Figure 7A,B).

When grouped by obese vs. non-obese, a significant correlation was found in both groups, but only in men, not in women (Figure S5, Supplementary Materials). In contrast, the mode and mean MFI indexes are both independent of SFT, as well as categorizations according to obese vs. non-obese, or male vs. female (Figure S6, Supplementary Materials).

#### 3.5.3. MFI Indexes’ Correlation with Age and BMI

**Figure 8:**
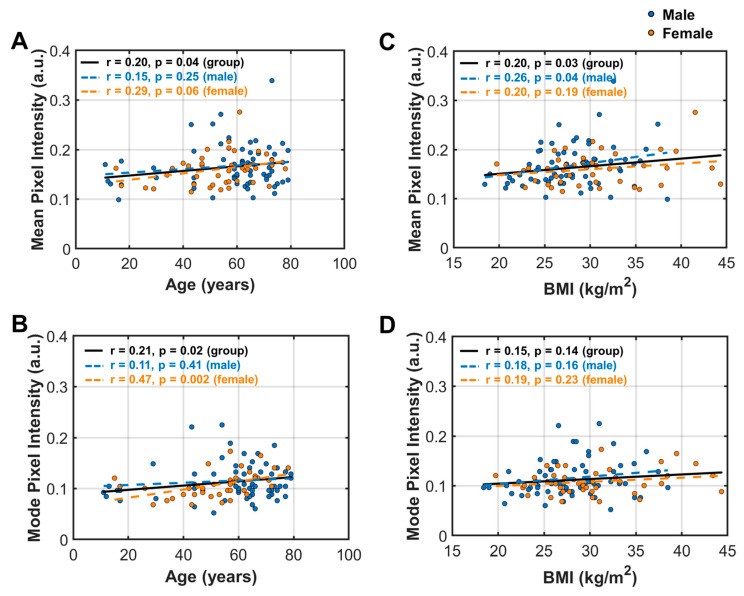
Analysis of linear correlation between muscle fat infiltration (MFI) indexes with demographic factors. (**A**) Age vs. mean pixel intensity; (**B**) BMI vs. mean pixel intensity; (**C**) age vs. mean pixel intensity; (**D**) BMI vs. mode pixel intensity.

For both mean and mode indexes, MFI tends to increase with age (Figure 8A,B), again with a sex disparity. In women, the mode index showed a stronger age correlation than the mean index (*p* = 0.002 vs. *p* = 0.06). In contrast, in men, age dependence was not found in either the mode or mean MFI index (*p* > 0.05). No difference in these sex disparities was noticed for the age dependence between obese vs. non-obese groups (Figure S7, Supplementary Materials).

However, a different sex disparity was observed in the MFI’s correlation with BMI. The mean MFI index is significantly correlated with BMI in men (*p* = 0.04), but not in women (*p* = 0.19, Figure 8C). In contrast, the mode MFI index shows no correlation with BMI in either men or women (Figure 8D).

Figure S8 (Supplementary Materials) illustrates the MFI multivariate regression with both age and BMI. For both mean (Figure S8A–C) and mode (Figure S8D–F) MFI indexes, the severity of MFI tends to increase with age and BMI. However, statistical significance was observed only within the female subgroup (*p* = 0.03 for mean pixel intensity, Figure S8C; and *p* < 0.01 for mode pixel intensity, Figure S8F), not in the male subgroup (*p* > 0.05 Figure S8B,E).

Tables S1–S4 (Supplementary Materials) summarizes the results of a multivariate correlation analysis of SFT, BMA, and MFI for the entire group and the male and female subgroups, with and without correction for the contribution of covariates. In brief, the above-mentioned sex disparity in fat distribution still holds after the correction of covariates.

### 3.6. Measurement Variations

Measurement variations from repeated manual ROI segmentations on the same image were averaged at 3.0% for SFT, 3.4% for BMA, 3.2% for the MFI mean index, and 4.1% for the MFI mode index, which are approximately 10-20-fold smaller than the corresponding inter-subject measurement variations: 60.5% for SFT, 60.4% for BMA, and 24.1% and 29.0% for the MFI mean and mode indexes, respectively. No alternations in correlation significance were found when the segmentation variations were introduced as random noise in SFT, BMA, and MFI measurements to assess their correlations with age and BMI.

## 4. Discussion

This study characterized the fat infiltration in calf muscle and fat distribution in subcutaneous tissue and bone marrow. For the entire group (*n* = 107 subjects), we found an opposite pattern between SFT and BMA in terms of their correlation with age. SFT tends to decrease with age (Figure 3A), whereas BMA tends to increase with age (Figure 4A). Notably, the age dependence of SFT is statistically significant only in women, not in men, whereas the age dependence of BMA is statistically significant only in men, not in women. This finding highlights a strong sex effect in fat distribution during aging. In other words, these observations suggest a distinct role of subcutaneous tissue versus bone marrow in storing fat and regulating fat metabolism in men versus women during their life span [31].

We also found that MFI increases with age, as one would anticipate. However, this correlation is statistically significant only in women, not in men (Figure 8A,B), with a higher sensitivity shown in the mode compared to the mean MFI index (*p* = 0.002 vs. *p* = 0.06). These MFI findings about age and sex dependences are consistent with early studies that have established that fat infiltration, which is detrimental to normal tissue functions, tends to worsen with aging in a variety of organs, such as the liver, heart, and skeletal muscles, and in women with estrogen deficiency [32,33,34,35,36,37,38].

In addition to age, BMI also affects MFI, but this is only statistically significant in men, not in women (Figure 8C), and is more significant with the mean index than the mode index (Figure 8C,D), which contrasts with the age dependence mentioned above. Such distinct sex effects on MFI, i.e., women being more sensitive to ageing while men are more sensitive to the effects of weight, call for sex-based strategies to reduce MFI.

Group analysis reveals that obese and non-obese men differ in their correlation between SFT and age (Figure S2), as well as between MFI and BMA (Figure S5, Supplementary Materials). The strong correlation between SFT and BMI observed in women and not in men (Figure 3B) suggests that the female calf SFT may serve as a sensitivity index for obesity, providing an alternative to BMI. Indeed, the seven participants with the highest SFTs (>12 mm) were all females. Of them, six were obese and one was overweight (Figure 4B).

Another novel finding of this study is that MFI is significantly correlated with BMA (Figure 7A,B). To the best of our knowledge, an association between fibular BMA and calf MFI has not been reported in the literature to date, although a number of factors that may trigger MFI and/or increase BMF have been suggested, including aging, disuse, metabolic syndromes/diseases, non-metabolic diseases, and muscle injury for MFI [36,37], and aging, estrogen deficiency, mechanical unloading, and exposure to glucocorticoids for BMF [38]. Notably, MFI is correlated with BMA but not SFT (Figure 7C,D), even though the calf muscle is physically attached to subcutaneous fat tissue while being separated by the fibular bone.

Is there a cross-talk between the fibular BMF and the fat in the calf muscle? In view of recent studies showing that bone marrow participates in central nervous system inflammation and autoimmunity [39], and that bone marrow channels in the skull serve as immune gateways to the central nervous system and drive the progression of multiple sclerosis [40,41,42], we speculate that the observed positive correlation between MFA and MFI (Figure 7) may reflect a similar metabolic regulation pathway between the fibula bone marrow and the fat infiltration into the calf muscle. A healthy muscle is known to rely on adequate bone marrow for the production of blood components to support muscle function, prevent infection, and control bleeding. Conversely, a healthy bone marrow relies on the normal functions of its supporting body parts, including bone and skeletal muscle, as evidenced by the fact that clinical bone marrow conditions often manifest as muscle symptoms such as muscle weakness and fatigue [43].

In terms of the cellular and molecular mechanisms, inflammation may be a shared contributor to the increased BMF and MFI. It has been proposed that bone marrow cell activity may be subject to modulation by inflammatory stimuli that originate remotely in inflamed peripheral organs through the bloodstream [44]. Consistent with the findings of a positive correlation between age and MFI severity (Figure 8), early studies have shown that MFI is prevalent among older adults [45] and that inflammation in aging is associated with EMCL accumulation and the subsequent insulin insensitivity [46]. On the other hand, the observation of a positive correlation between age and BMA, especially in women (Figure 7), is consistent with the early findings that bone marrow volume increases with aging and in osteoporosis [38,46,47], and that inflammation is a contributor to both osteoporosis and the expansion of adipose tissues [47,48,49].

Thus, the MFI progression and increase in BMF volume may be linked to inflammation and gradual loss of bone density or osteoporosis—conditions that affect 10% of women versus 2% men over the age of 50 in the US [50,51]. Osteoporosis in association with muscle weakness is a main reason for falls and bone fractures [52], contributing to >500,000 hospitalizations, 800,000 emergency room visits, 2.6 million physician office visits, and 180,000 nursing home placements annually in the US [50]. This demands effective strategies to strengthen bone and skeletal muscle, especially the fibular bone and the surrounding calf muscle, which play a crucial role in supporting the body’s balance against falling. While strength exercises can increase bone density and lean muscle mass, other interventions, such as vibration therapy and sleeve gastrectomy, may also be needed in patients with conditions such as exercise intolerance and obesity [53,54]. Non-invasive vibration therapy was recommended by the International Society of Musculoskeletal and Neuronal Interactions to improve both bone density and muscle strength, probably through releasing muscle stress, stimulating circulation, or neuromuscular activation [50,55]. Inhibiting marrow adipogenesis may be another therapeutic possibility to prevent or treat bone density loss [56].

There are several limitations of this work. Firstly, the study group was heterogeneous in BMI, with the average tending toward the overweight category (BMI: men 28.4; women 30.3; among them, heathy weight 20%, overweight 44%, and obese 36%), although this is reflective of the average US adult population (BMI: men 29.1 and women 29.6; healthy weight: men 27% and women 36%; overweight: men 41% and women 30%; obese: men 31% and women 33%) [57,58]. Secondly, although accurate anatomical data can be obtained at routine 1.5T or 3T [59], this study was based on available MRI data at ultrahigh field 7T, which is being increasingly used due to its intrinsically high signal-to-noise ratio and image contrast between fat and lean muscles, which make it useful for the accurate quantification of SFT, BMA, and MFI [60,61]. Thirdly, the effect of leg dominance was not systematically studied due to limited data availability. Of the three subjects scanned on both the left and right legs, no statistically significant difference was found in SFT and BMA between the left and right legs (Figure S9, Supplementary Materials), which warrants further study with large groups in the future. Finally, this study evaluated marrow fat in the fibula, whereas the tibia was not evaluated. This is because the fibula, with a higher cross-sectional homogeneity on MRI images and a more consistent BMA along the FH direction, is directly attached to the ROI calf muscle. In contrast, the tibia is away from the periphery calf muscle ROI and suffers from the issue of a fairly large cross-sectional inhomogeneity and BMA gradient along the FH direction (Figure S1, Supplementary Materials).

Combined, our findings on MFI in relation to age, BMA, SFT, and BMI may offer novel insights into the MFI mechanism and contribute to the prevention and treatment of MFI-associated muscle dysfunctions. More specifically, our results may suggest that exercise interventions targeting subcutaneous adiposity in the lower extremities may offer greater benefits to women, particularly the elderly, compared to men. This insight underscores the importance of tailored interventions that are based on sex and age for effectively promoting muscle health and overall well-being.

## Data Availability

Data in this study are available upon request to the corresponding authors.

## Supplementary Materials

The following supporting information can be downloaded at: https://www.mdpi.com/article/10.3390/diagnostics14202260/s1, Figure S1. Axial (left panel) and sagittal (right penal) T2w MR images showing consistency in cross-sectional area and mage homogeneity along FH direction in fibula bone marrow, in contrast to tibia bone marrow with large variations. Figure S2. Linear correlation of age with SFT for non-obese (A, n = 69) and obese (B, n = 38, BMI > 30) groups. Figure S3. Linear correlation of age with BMA for non-obese (A, n = 69) and obese (B, n = 38, BMI > 30) groups. Figure S4. Analysis of pixel histogram for characterization of the severity of fat infiltration in calf muscle. (A) Pixel intensity distribution profiles, showing mean pixel intensity (black dash line) and mode pixel intensity (magenta dash line). (B) Subject clustering based on the measurements of pixel mean intensity alone. Muscle fat infiltration (MFI) in 107 subjects clustered into four groups, normal (44/107, 27M/17F), mild MFI 37/107, 20M/17F), moderate MFI (23/107, 15M/7F) and severe MFI (3/107, 2M/1F). Note the trend of MFI reflected by the increased mean intensity, mode intensity and linewidth (profile dispersion). Figure S5. Linear correlation of BMA with MFI mean (A and B) and mode (C and D) indexes for non-obese (A and C, n = 67) and obese (B and D, n = 38, BMI > 30) groups. Figure S6. Linear correlation of SFT with MFI mean (A and B) and mode (C and D) indexes for non-obese (A and C, n = 67) and obese (B and D, n = 38, BMI > 30) groups. Figure S7. Linear correlation of age with MFI mean (A and B) and mode (C and D) indexes for non-obese (A and C, n = 67) and obese (B and D, n = 38, BMI > 30) groups. Figure S8. Multivariate regression analysis between muscle fat infiltration (MFI) indexes with age and BMI (n = 105, no critical fat infiltration cases). All subjects (A) and (D); men subgroup (B) and (E); women subgroup (C) and (F). Figure S9. Multivariate regression analysis between muscle fat infiltration (MFI) indexes with age and BMI (n = 105, no critical fat infiltration cases). All subjects (A) and (D); men subgroup (B) and (E); women subgroup (C) and (F). Table S1. r- and *p*-values for BMA and SFT correlations with age, with and without correction for the BMI effect; Table S2. r- and *p*-values for BMA and SFT correlations with BMI, with and without correction for the age effect; Table S3. r- and *p*-values for MFI correlations with age, with and without correction for the BMI effect (w/o critical fat infiltration cases); Table S4. r- and *p*-values for MFI correlations with BMI, with and without correction for the age effect (w/o critical fat infiltration cases).
