# Supplementary material for: Sex Differences in Fat Distribution and Muscle Fat Infiltration in the Lower Extremity: A Retrospective Diverse-Ethnicity 7T MRI Study in a Research Institute Setting in the USA
Source: Diagnostics (Basel). 2024 Oct 10;14(20):2260. doi: 10.3390/diagnostics14202260 (PMC11506611; doi:10.3390/diagnostics14202260)
Supplement: Supplementary file 1 [file diagnostics-14-02260-s001.zip › diagnostics-3131425-supplementary.pdf]

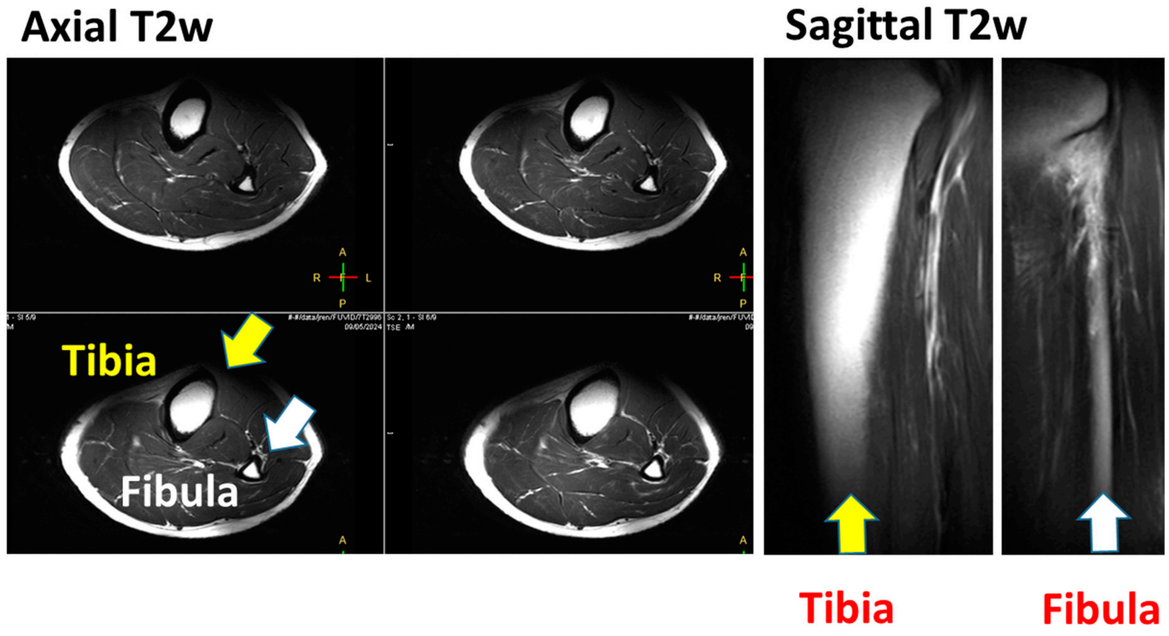

Figure S1. Axial (left panel) and sagittal (right penal) T2w MR images showing consistency in cross-sectional area and mage homogeneity along FH direction in fibula bone marrow, in contrast to tibia bone marrow with large variations.

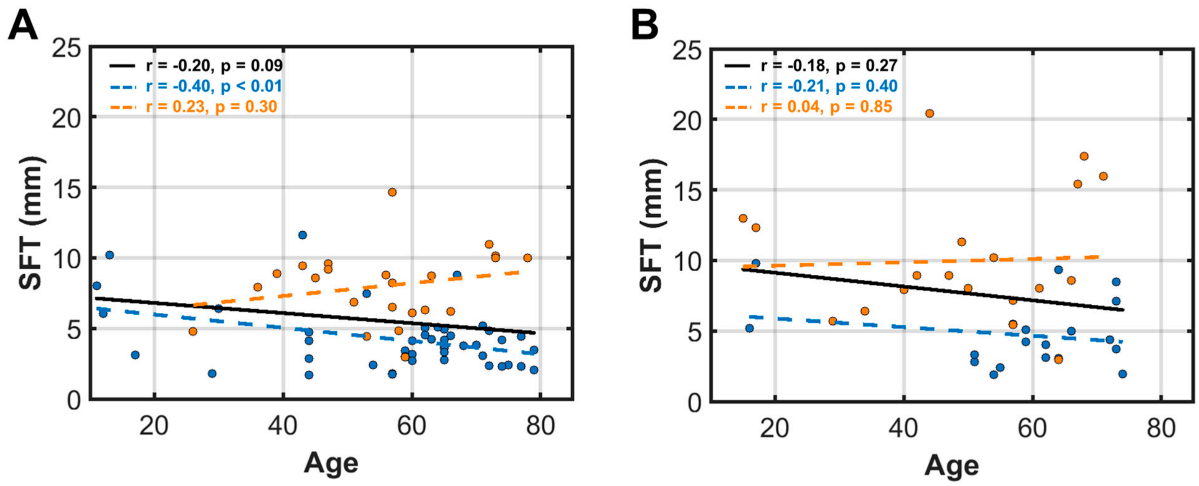

Figure S2. Linear correlation of age with SFT for non-obese (**A**, n = 69) and obese (**B**, n = 38, BMI > 30) groups.

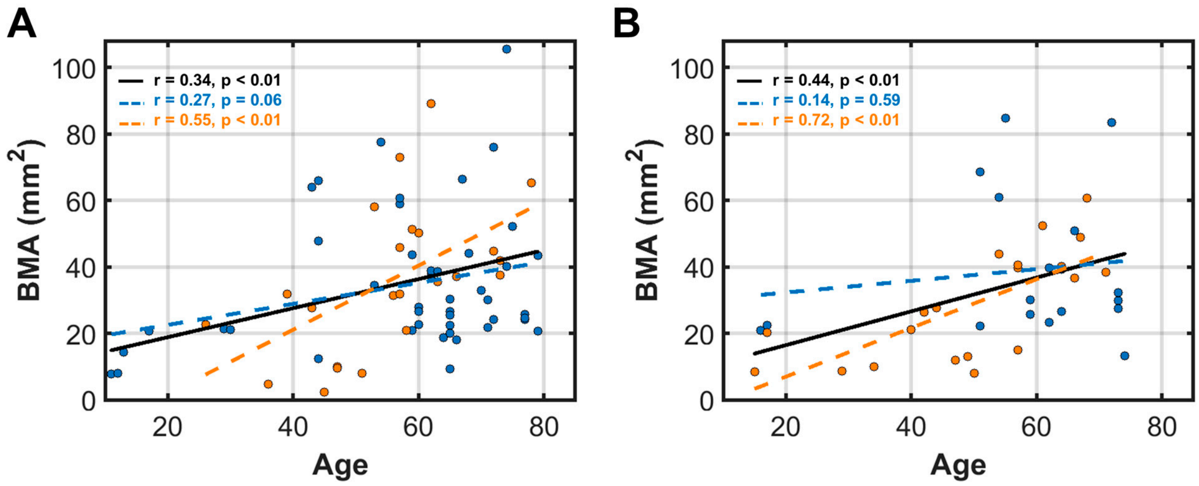

Figure S3. Linear correlation of age with BMA for non-obese (**A**, n = 69) and obese (**B**, n = 38, BMI > 30) groups.

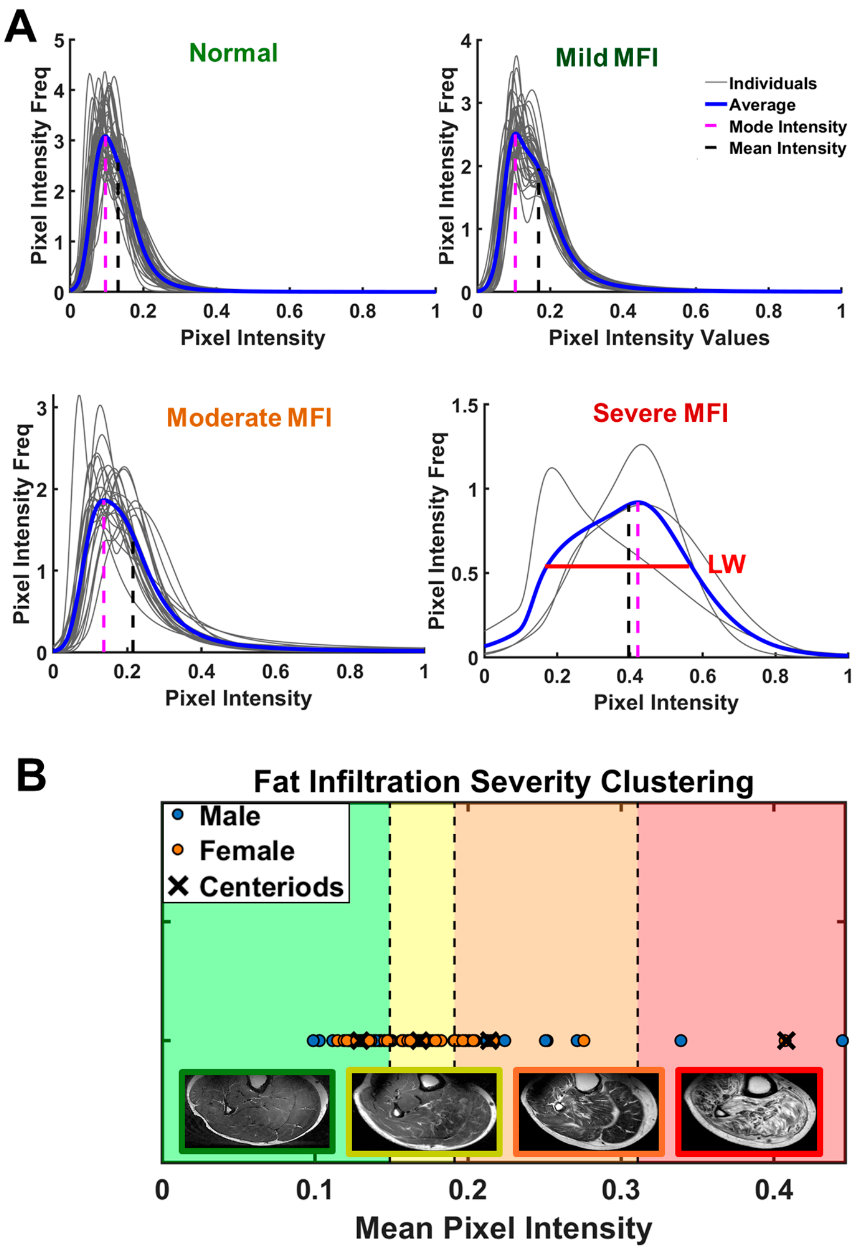

Figure S4. Analysis of pixel histogram for characterization of the severity of fat infiltration in calf muscle. (A) Pixel intensity distribution profiles, showing mean pixel intensity (black dash line) and mode pixel intensity (magenta dash line). (B) Subject clustering based on the measurements of pixel mean intensity alone. Muscle fat infiltration (MFI) in 107 subjects clustered into four groups, normal (44/107, 27M/17F), mild MFI 37/107, 20M/17F), moderate MFI (23/107, 15M/7F) and severe MFI (3/107, 2M/1F). Note the trend of MFI reflected by the increased mean intensity, mode intensity and linewidth (profile dispersion).

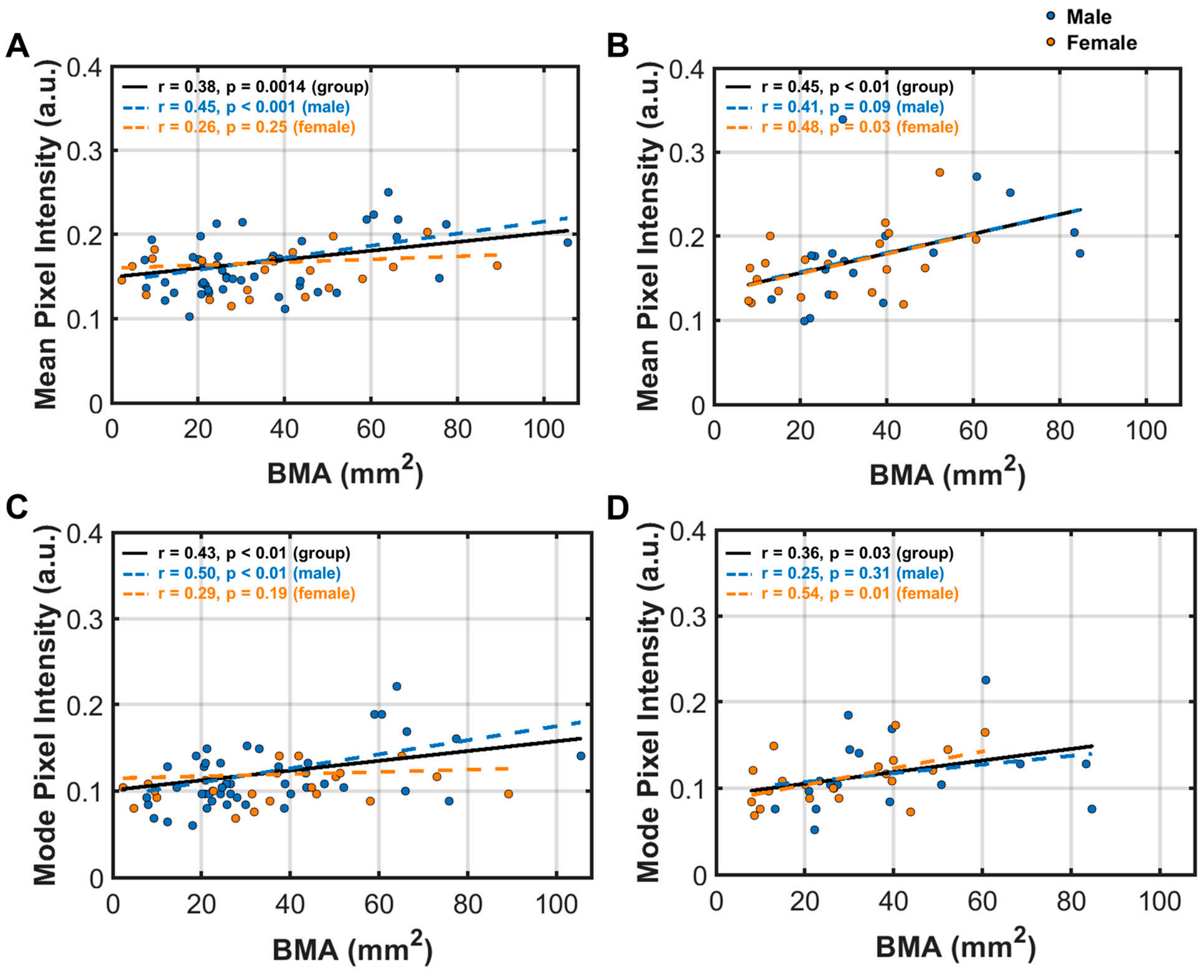

Figure S5. Linear correlation of BMA with MFI mean (**A** and **B**) and mode (**C** and **D**) indexes for non-obese (**A** and **C**, n = 67) and obese (**B** and **D**, n = 38, BMI > 30) groups.

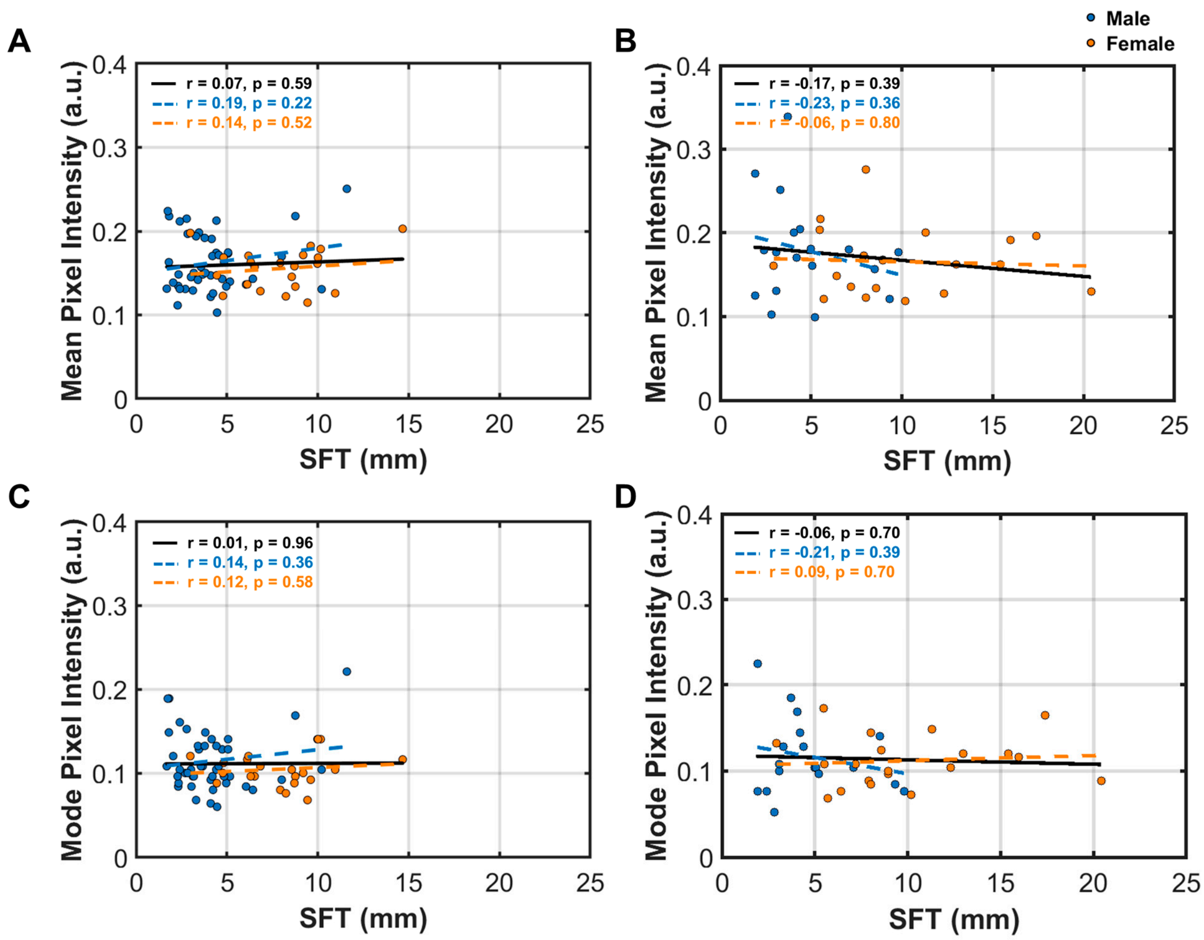

Figure S6. Linear correlation of SFT with MFI mean (**A** and **B**) and mode (**C** and **D**) indexes for non-obese (**A** and **C**, n = 67) and obese (**B** and **D**, n = 38, BMI > 30) groups.

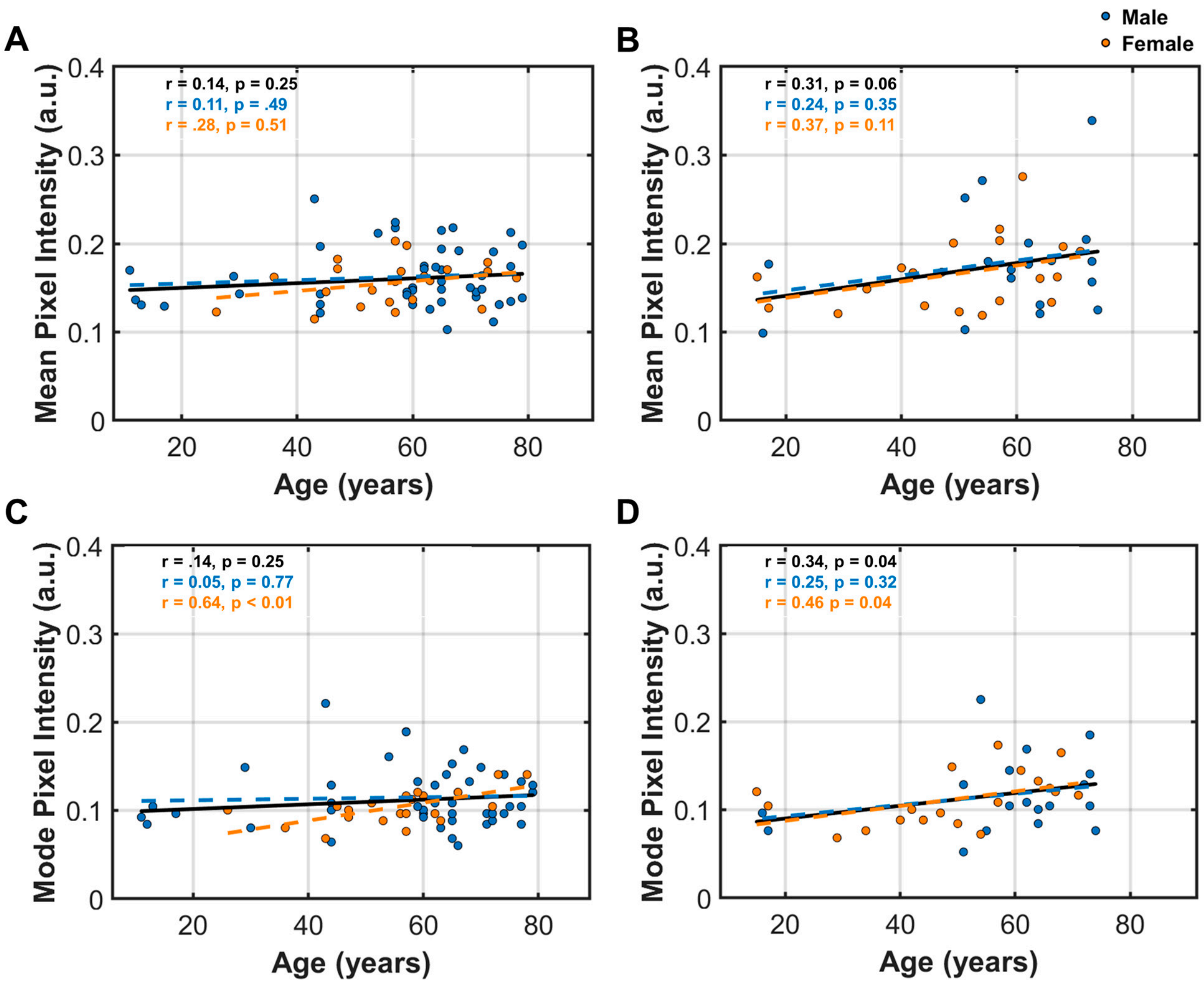

Figure S7. Linear correlation of age with MFI mean (**A** and **B**) and mode (**C** and **D**) indexes for non-obese (**A** and **C**, n = 67) and obese (**B** and **D**, n = 38, BMI > 30) groups.

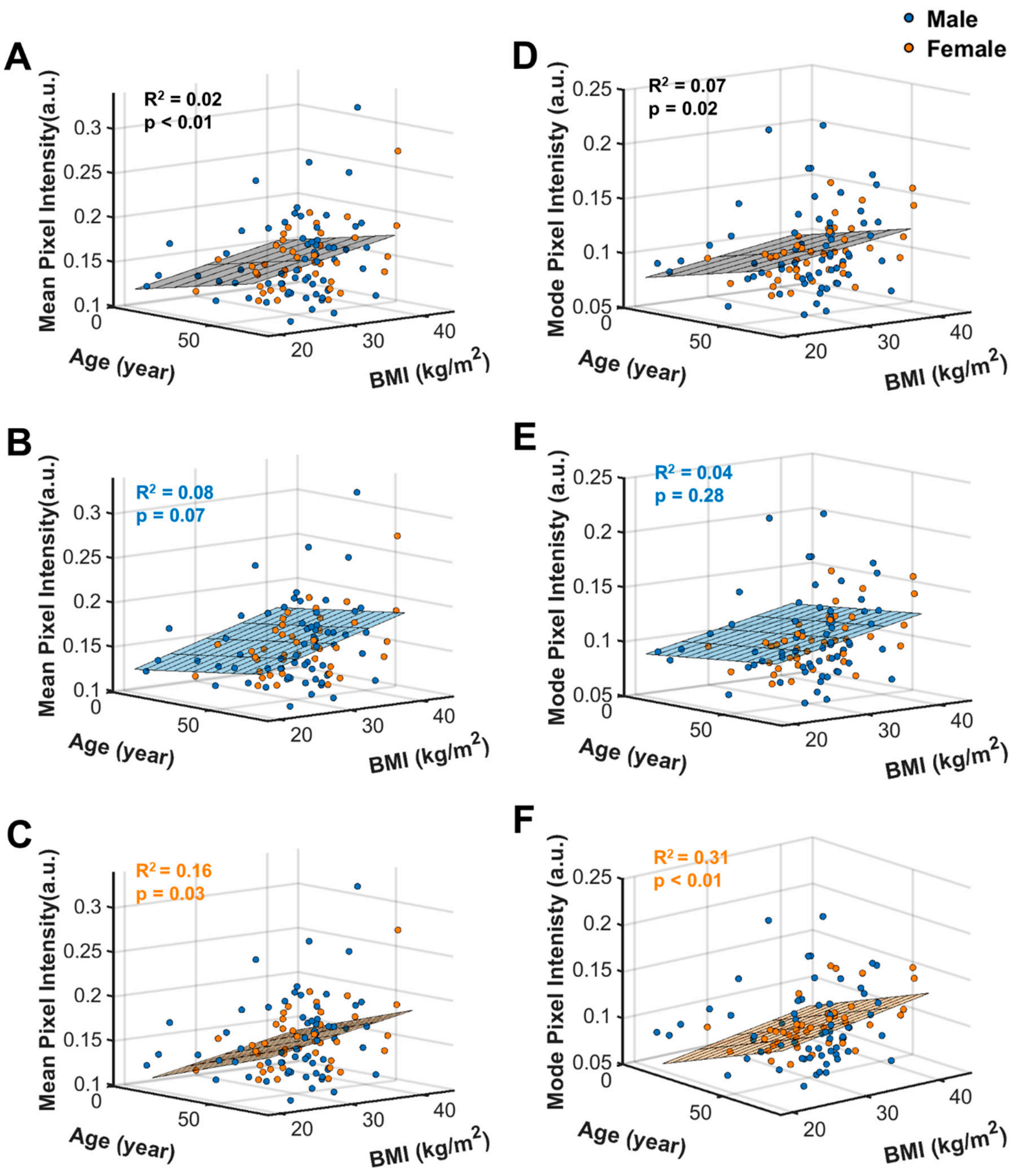

Figure S8. Multivariate regression analysis between muscle fat infiltration (MFI) indexes with age and BMI (n = 105, no critical fat infiltration cases). All subjects (A) and (D); men subgroup (B) and (E); women subgroup (C) and (F).

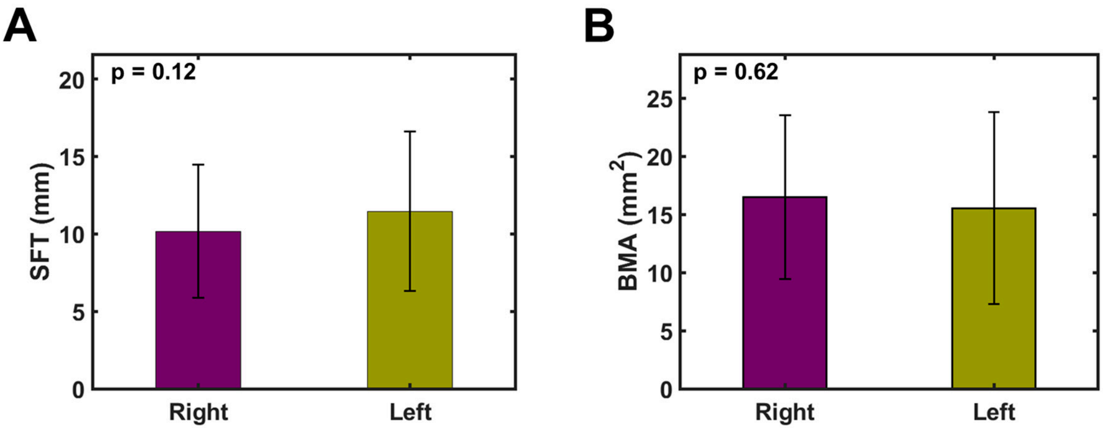

Figure S9. Multivariate regression analysis between muscle fat infiltration (MFI) indexes with age and BMI (n = 105, no critical fat infiltration cases). All subjects (A) and (D); men subgroup (B) and (E); women subgroup (C) and (F).

Table S1. r- and p-values for BMA and SFT correlation with age with and without correction for the BMI effect

	BMA				SFT			
	age (raw)		age (correction)		age (raw)		age (correction)	
Subject Groups	r	p	r	p	r	p	r	p
Male (n = 64)	0.24	0.06	0.23	0.07	-0.34	0.0059	-0.35	0.0047
Female (n = 43)	0.62	<0.0001	0.63	<0.0001	0.04	0.79	0.21	0.17
Group (n = 107)	0.37	<0.0001	0.39	<0.0001	-0.21	0.03	-0.19	0.048

Table S2. r- and p-values for BMA and SFT correlation with BMI with and without correction for the age effect

	BMA				SFT			
	BMI (raw)		BMI (correction)		BMI (raw)		BMI (correction)	
Subject Groups	r	p	r	p	r	p	r	p
Male (n = 64)	0.25	0.04	0.24	0.05	0.09	0.47	0.12	0.35
Female (n = 43)	-0.07	0.65	0.08	0.59	0.57	<0.0001	0.60	<0.0001
Group (n = 107)	0.08	0.39	0.12	0.19	0.43	<0.0001	0.42	<0.0001

Table S3. r- and p-values for MFI correlation with age with and without correction for the BMI effect (w/o critical fat infiltration cases)

	Mean PI				Mode PI			
	age (raw)		age (correction)		age (raw)		age (correction)	
Subject Groups	r	p	r	p	r	p	r	p
Male (n = 63)	0.15	0.25	0.14	0.29	0.11	0.41	0.10	0.44
Female (n = 42)	0.29	0.06	0.36	0.02	0.47	0.002	0.54	0.0002
Group (n = 105)	0.20	0.04	0.22	0.02	0.21	0.03	0.23	0.02

Table S4. r- and p-values for MFI correlation with BMI with and without correction for the age effect (w/o critical fat infiltration cases)

	Mean PI				Mode PI			
	BMI (raw)		BMI (correction)		BMI (raw)		BMI (correction)	
Subject Groups	r	p	r	p	r	p	r	p
Male (n = 63)	0.25	0.04	0.25	0.045	0.18	0.16	0.18	0.17
Female (n = 42)	0.20	0.19	0.30	0.056	0.19	0.23	0.35	0.02
Group (n = 105)	0.20	0.04	0.23	0.02	0.15	0.14	0.17	0.08
